# Comprehensive real time remote monitoring for Parkinson’s disease using Quantitative DigitoGraphy

**DOI:** 10.1038/s41531-024-00751-w

**Published:** 2024-07-27

**Authors:** Shannon L. Hoffman, Paul Schmiedmayer, Aryaman S. Gala, Kevin B. Wilkins, Laura Parisi, Shreesh Karjagi, Aarushi S. Negi, Simon Revlock, Christopher Coriz, Jeremy Revlock, Vishnu Ravi, Helen Bronte-Stewart

**Affiliations:** 1 Department of Neurology and Neurological Sciences, Stanford School of Medicine, Stanford, CA USA; 2 Stanford Byers Center for Biodesign, Stanford University, Stanford, CA USA; 3 Fastlane Creations, Encinitas, CA USA; 4 Stanford Medicine Catalyst, Stanford School of Medicine, Stanford, CA USA; 5 Department of Neurosurgery, Stanford School of Medicine, Stanford, CA USA

**Keywords:** Parkinson's disease, Parkinson's disease, Physical examination, Parkinson's disease, Neurological manifestations

## Abstract

People with Parkinson’s disease (PWP) face critical challenges, including lack of access to neurological care, inadequate measurement and communication of motor symptoms, and suboptimal medication management and compliance. We have developed QDG-Care: a comprehensive connected care platform for Parkinson’s disease (PD) that delivers validated, quantitative metrics of all motor signs in PD in real time, monitors the effects of adjusting therapy and medication adherence and is accessible in the electronic health record. In this article, we describe the design and engineering of all components of QDG-Care, including the development and utility of the QDG Mobility and Tremor Severity Scores. We present the preliminary results and insights from an at-home trial using QDG-Care. QDG technology has enormous potential to improve access to, equity of, and quality of care for PWP, and improve compliance with complex time-critical medication regimens. It will enable rapid “Go-NoGo” decisions for new therapeutics by providing high-resolution data that require fewer participants at lower cost and allow more diverse recruitment.

## Introduction

Parkinson’s disease (PD) is the fastest growing neurological disorder and has been called an emerging pandemic^1^. People with PD (PWP) must take multiple time-critical medications throughout the day to maintain dopamine levels in affected brain networks. Optimizing such complicated medication schedules to provide personalized care with actionable insights requires management by a neurologist; yet only 2% of physicians become neurologists and over 40% of PWP do not have access to neurological care^2–5^. Even for PWP who do have access to a neurologist, there are usually three to six month intervals between in-person visits and there is no remote, real-time, comprehensive system to monitor the effects of therapy and/or symptom progression. The Movement Disorders Society-Unified Parkinson’s Disease Rating Scale motor examination (MDS-UPDRS III) is the only standardized way to document the severity of motor signs, which requires an in-person evaluation. However, only fellowship trained movement disorders specialists (0.14% of physicians) receive in depth training in the MDS-UPDRS and this has further limited the supply of physicians who feel comfortable managing PD.

Communicating unanticipated problems by the PWP to the health care team is currently inefficient and relies on verbal descriptions of complex movement signs; the provider has to decide whether to make medication adjustments based on such information or whether to squeeze the person on to an over-capacity clinic schedule. These critical deficiencies in the care of PWP lead to suboptimal medication management and compliance, which have resulted in increased numbers of emergency room (ER), hospital, and ICU admissions with a longer length of stay than age-matched non-PD patients^6–8^. This has resulted in a total economic burden for the care of PWP in the US of over $51 billion annually^9^.

Another major deficiency is the lack of high-resolution quantitative measurements of motor signs in PD that can be used as outcomes in multicenter pivotal clinical trials, which may have contributed to the paucity of novel therapeutics that have emerged for PD, especially in early or prodromal disease stages. The MDS-UPDRS III suffers from inter- and intra-rater variability^10^. Its use as the primary outcome of multicenter trials has several problems: the variability in rating has required a single certified rater to rate video recordings of the MDS-UPDRS III and this results in the loss of any assessment of rigidity; the ability to detect statistical differences in motor function using such a scale has required large cohorts, who have to travel to a center for in person evaluations; and there is substantial difficulty in recruiting adequate numbers of participants for such trials and in achieving diversity in the participant pool.

Finally, the Covid-19 pandemic suddenly cut PWP off from seeing their providers in person and led to the same difficulties of using video only assessments during telemedicine visits. This highlighted the need for a fully connected care platform that could give the provider accurate comprehensive motor assessments and information about adherence to medication schedules in a remote context to facilitate actionable clinical insights and be integrated into telemedicine.

Previously we introduced a novel digital technology called Quantitative Digitography (QDG), which provides quantitative, validated metrics of all motor signs of PD in real-time from thirty seconds of repetitive alternating finger tapping (RAFT) on adjacent tensioned engineered levers^11–18^. QDG-RAFT metrics have been validated against the UPDRS III, the MDS-UPDRS III and its subscores, including rigidity, which is currently unavailable from video or any wearable sensor; QDG metrics are correlated with both upper and lower extremity MDS-UPDRS III subscores and with gait impairment and freezing of gait^12,17,18^. QDG monitors the effect of therapy and the progression of disease^11,12,17^. QDG also distinguishes motor abnormalities in untreated very early-stage PD, and RAFT detects those with idiopathic REM sleep behavior disorder (iRBD), who would convert to PD earlier than the UPDRS III^19,20^.

**Fig. 1:**
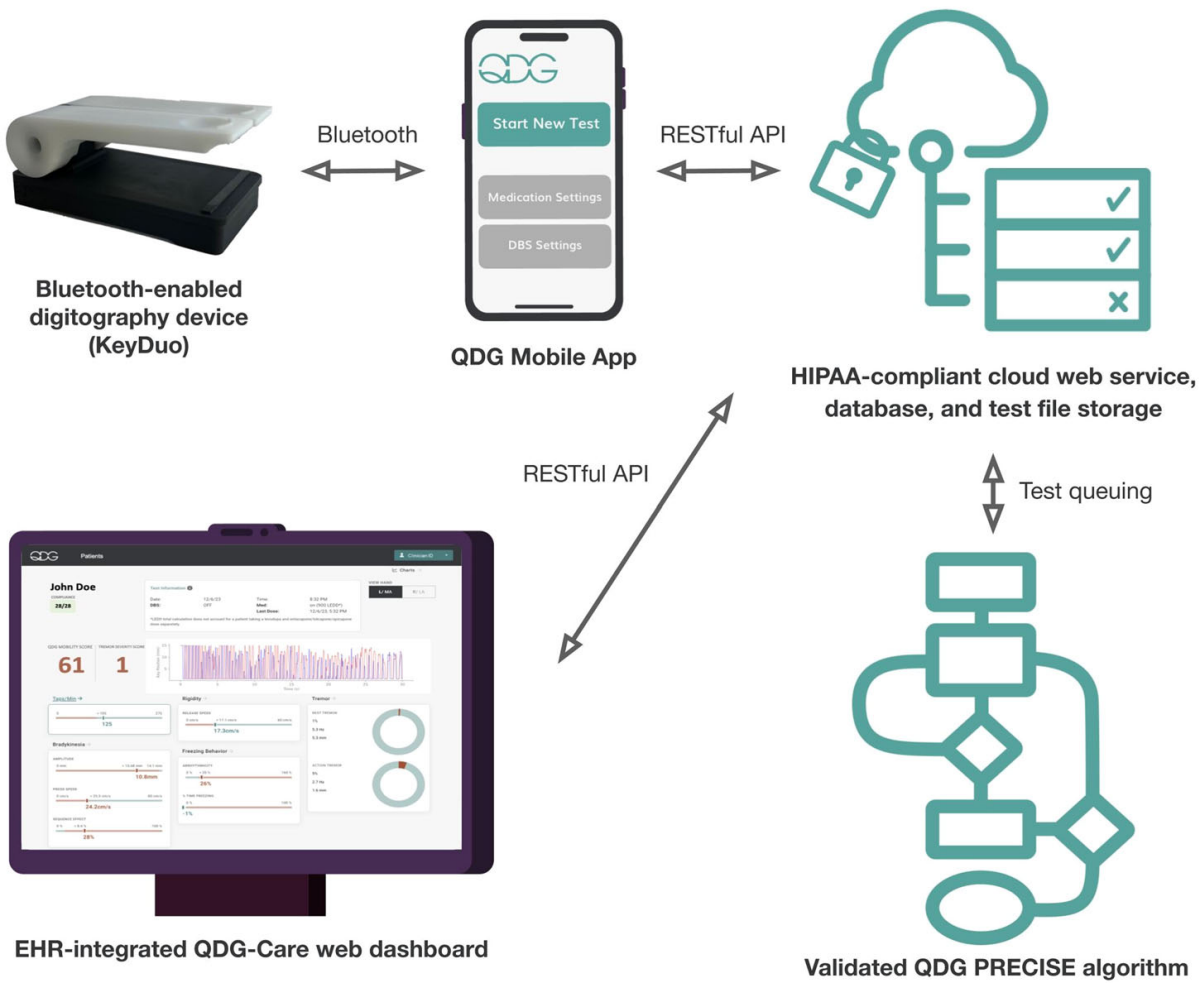
Overview of the QDG-Care platform demonstrating the data flow in the system. The RAFT test is performed on the KeyDuo device and raw keypress position data is transferred to the mobile application using Bluetooth. The mobile application performs a device calibration-specific data transformation of raw measurements, initial error screening, and medication and DBS settings management. The data is synced and retrieved from the HIPAA-compliant cloud web service managing file storage and a database. Each test is enqueued in an algorithm queue to be analyzed by a versioned QDG PRECISE algorithm returning the processed information, including the Mobility and Tremor Severity Scores and other metrics, to the web service. The resulting data is shown within the EHR using a SMART-on-FHIR Dashboard, providing physicians real-time access to the quantitative data.

In this article, we will detail the development of a fully integrated connected care platform (QDG-Care) consisting of a high precision, Bluetooth-enabled digitography device (KeyDuo), a patient-facing mobile application with local processing, a HIPAA-compliant cloud web service and customized algorithm (PRECISE), a provider dashboard, and electronic health record (EHR) integration, Fig. 1.

The integrated system was designed to meet the needs of PWP and providers by delivering remotely collected, validated, comprehensive metrics of the PWP’s motor symptoms and therapeutic state in real time. QDG provides flexible data capture (e.g., daily and as many times per day as needed) and captures therapeutic information (medication adherence, DBS settings) at and around the time of the QDG test. We will highlight the human factors motivating the design and engineering of the KeyDuo, the development of the QDG Mobility and Tremor Severity Scores and their utility in different use cases, and the development of the mobile app, cloud-based database, algorithm service, and dashboard. Finally, we will report the preliminary results of the pilot clinical trial of the fully integrated QDG-Care system, whose primary outcome was compliance with daily QDG-RAFT testing from home over 30 days. We will also highlight several scenarios from the trial where valuable clinical insights were derived from remote testing that would have otherwise been unknown or unavailable within standard clinical practice.

QDG-Care is a real-time comprehensive connected care platform for PD that delivers validated, quantitative metrics of all motor signs in PD in real time, monitors medication adherence and the effects of adjusting therapy, and has been integrated into the EHR. We hope that QDG will improve the access to, equity of, and quality of care for people with Parkinson’s disease and will improve compliance with and monitoring of complex time critical medication regimens. QDG’s high resolution data will allow rapid “Go-NoGo” decisions of potential therapeutics, requiring fewer participants at lower cost, and allowing remote recruitment and assessment.

## Results

A better understanding of motor behavior between visits is a frequently expressed need for both PWP and providers, particularly when PWP start or modify a therapeutic intervention, experience significant variability in symptoms throughout the day, or feel their symptoms present differently at home versus in the clinic (PWP and provider feedback). The QDG system needed to be able to present comprehensive metrics of motor function relative to control values, within the context of the patient’s therapeutic state, and at flexible intervals. The following sections will describe how these overarching needs, other specific input from patient and provider users, and technical requirements informed the design of each system component.

### The QDG KeyDuo

The QDG KeyDuo was designed with the patient user experience as a priority, while still achieving the technical requirements of the algorithm to obtain QDG metrics. The QDG-RAFT task was originally developed on a musical instrument digital interface (MIDI) keyboard, later employed on an engineered set of levers that were part of the At Home Testing Device (AHTD) developed in collaboration with Intel’s Digital Health Technology Group^13^, and subsequently developed in a lab prototype that has been used in research for over 14 years^11–19,21,22^. The KeyDuo consists of two adjacent tensioned levers, each with a highly accurate and precise sensor, spring, and controller board that were all selected to meet or exceed critical technical specifications of the earlier prototypes. The length of the levers creates the feeling of a natural arc to the movement, while also optimizing the accuracy of amplitude sensing. The sensors are positioned to avoid interaction with the adjacent sensor and minimize contamination from dirt or dust. The device is Bluetooth-enabled for automatic and wireless data transfer, and the charging port is placed in an easily accessible and expected location. All electronics and methods of sensing are hidden, allowing the user to focus on the task and not the device.

From the patient user’s perspective, the device needed to be small, portable, durable, and easy to use. As such, its dimensions (45 × 85 × 36 mm) allow the device to be easily carried in one hand and to be stored out in the open or in a drawer, as desired. The device weight (73 g) is also light enough to allow for easy transport, while still being slightly heavy for its size to minimize movement on the surface while in use as well as emphasize the user’s perception of its durability. Depressions with defined edges at the ends of the levers intuitively guide the user to the intended placement of the fingertips, allowing for standardized finger placement within and between tasks. Its curves and simple two-tone colors keep the user curious and intrigued, whereas a complex and single tone design could be viewed as intimidating, Fig. 1.

### The development of the QDG Mobility and Tremor Severity Scores

**Fig. 2:**
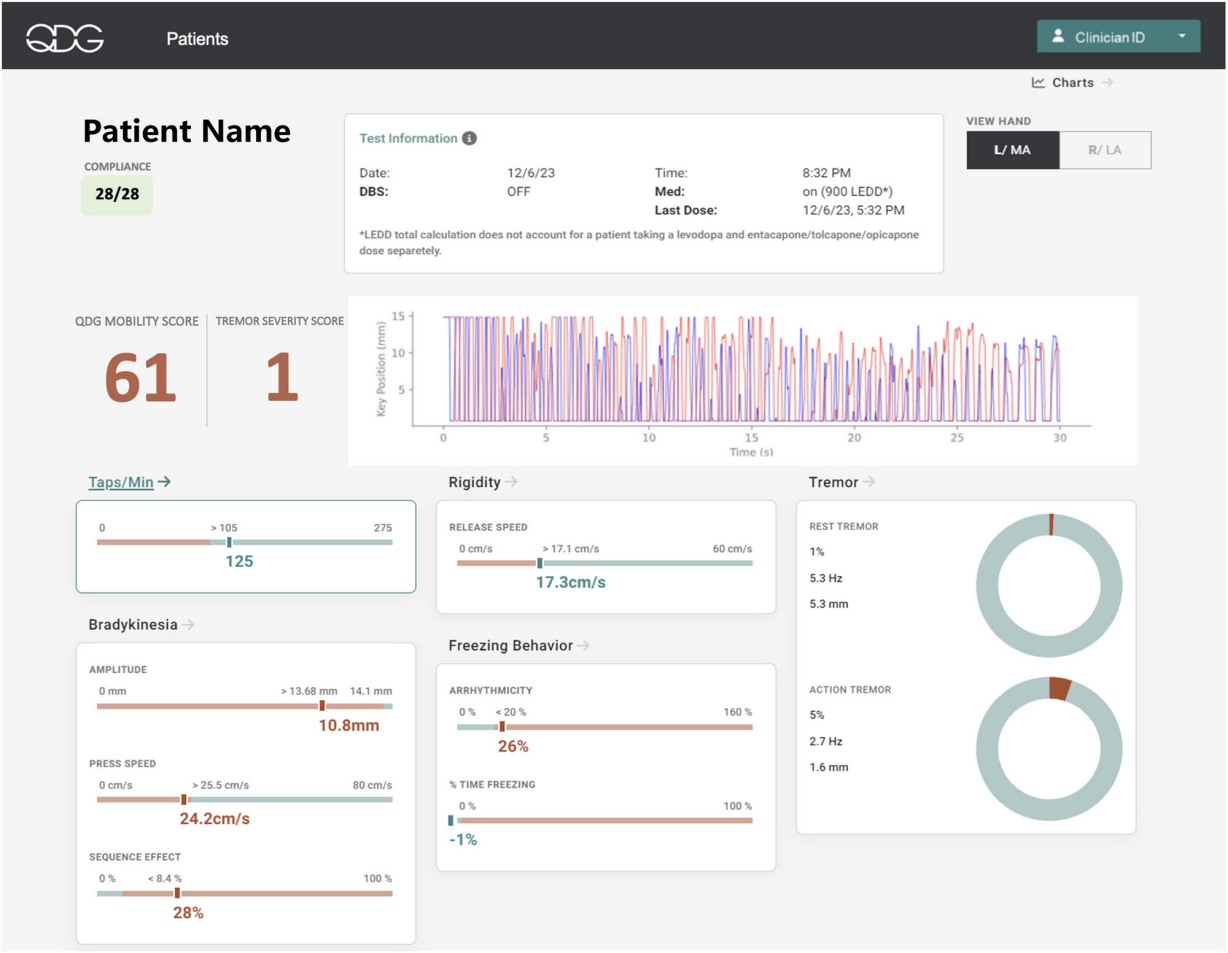
Depiction of the QDG web dashboard which visualizes the calculated metrics from a single RAFT test for all the cardinal motor signs of Parkinson’s Disease. The metrics displayed in green indicate that the values are within the range of age-matched healthy controls, whereas the values in red are outside the healthy control range.

Figure 2 depicts the visualized QDG metrics obtained from 30 seconds of RAFT on the KeyDuo. A single RAFT test yields four metrics of bradykinesia: number of taps per minute, mean press amplitude of taps, mean press speed and a metric reflective of the sequence effect in tap amplitude (the coefficient of variation of press amplitude). It also yields release speed, a validated metric of rigidity, rest and action tremor, and arrhythmicity, a metric correlated with gait impairment^12,15,17,18^. The number of days with a completed test divided by the total number of possible test days in the current month is represented by the Compliance ratio (top left in green). Details of the state of therapy (DBS and medication) are given along with the time of the last dose of medication (top center). Whether the left (L) or right (R) hand is the more affected (MA) or less affected (LA) is listed (top right). The QDG Mobility and Tremor Severity Scores are detailed below.

Providers desired a quick way to scan for deficits in mobility and tremor, with the ability to dive deeper into individual metrics as needed (provider feedback). The QDG Mobility Score was developed to provide a single score representing a participant’s overall movement proficiency by combining performance across different aspects of movement including speed, frequency, amplitude, and rhythmicity. It is represented by a single value between 0 and 100, where 100 represents ideal performance. This was accomplished by first establishing age-based distribution of healthy control performance across each of the relevant QDG metrics. A statistically-driven weighting of overall performance could then be determined from the observed QDG metrics in reference to the performance of an age-matched healthy control (see methods for additional details). Since tremor is a rhythmic, high-frequency involuntary movement, it can affect various QDG metrics, and therefore, needs to be excluded when measuring different aspects of voluntary movement. The algorithm excludes tremor identified in the trial on a strike-by-strike basis^18^, thus enabling QDG metrics to be quantified only on voluntary non-tremor strikes. The proportion and amplitude of tremor-identified strikes was used to provide a separate Tremor Severity Score (see methods for additional details) to complement the QDG Mobility Score.

In a cohort of 51 individuals with PD either off or on therapy tested in the clinic, the QDG Mobility Score was significantly negatively associated with the hemibody MDS-UPDRS III (sum of the axial and lateralized bradykinesia and rigidity items, β_0_ = −0.11, t= −6.26, *p* = 2.81e−09,). The β_0_ indicates that a 0.11 decrease in hemibody MDS-UPDRS III score is associated with a one unit increase in the QDG Mobility Score. Tremor sub-scores were excluded since the QDG Mobility Score was calculated using only voluntary strikes.

**Fig. 3:**
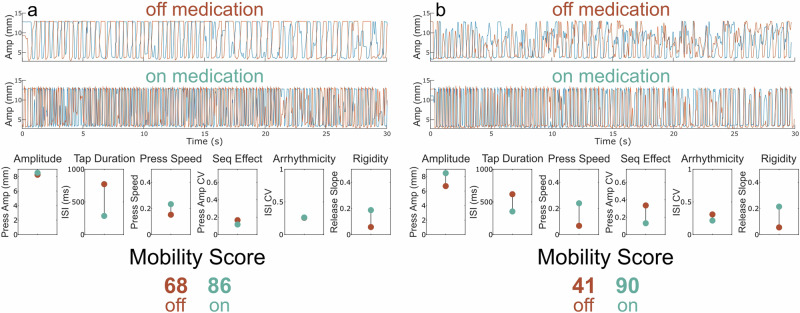
Examples of change in Mobility Score off versus on medication. (Top) Raw QDG trace for the index and middle finger off and (Middle) on medication. (Bottom) Performance across each of the six QDG metrics and the corresponding total Mobility Score with off medication depicted in red and on medication in green. Subtitles denote which aspect of movement is being measured. **a** Example of a QDG-RAFT trace off medication with large (close to normal) amplitude presses but a slow speed of tapping. On medication the speed of tapping (both the press speed and tap duration (ISI)) improved and this was reflected in an improvement of the QDG Mobility Score from 68 off medication to 86 on medication. **b** Example of QDG-RAFT trace off medication with slow press and release speeds, and low frequency. On medication, the mean press amplitude and speed of tapping improved, alongside improvements in sequence effect and rigidity, and the Mobility Score increased from 41 to 90.

Figure 3a shows a QDG-RAFT trace off medication with large (close to normal) amplitude presses but a slow speed of tapping. On medication the speed of tapping (both the press speed and tap duration (ISI)) improved and this was reflected in an improvement of the QDG Mobility Score from 68 off medication to 86 on medication. Another PWP had low, varied amplitude RAFT, with slow press and release speeds, and low frequency, off medication, Fig. 3b top and lowest panels. On medication, the mean press amplitude and speed of tapping improved, alongside improvements in sequence effect and rigidity, Fig. 3b middle and lowest panels, and the Mobility Score increased from 41 to 90.

**Fig. 4:**
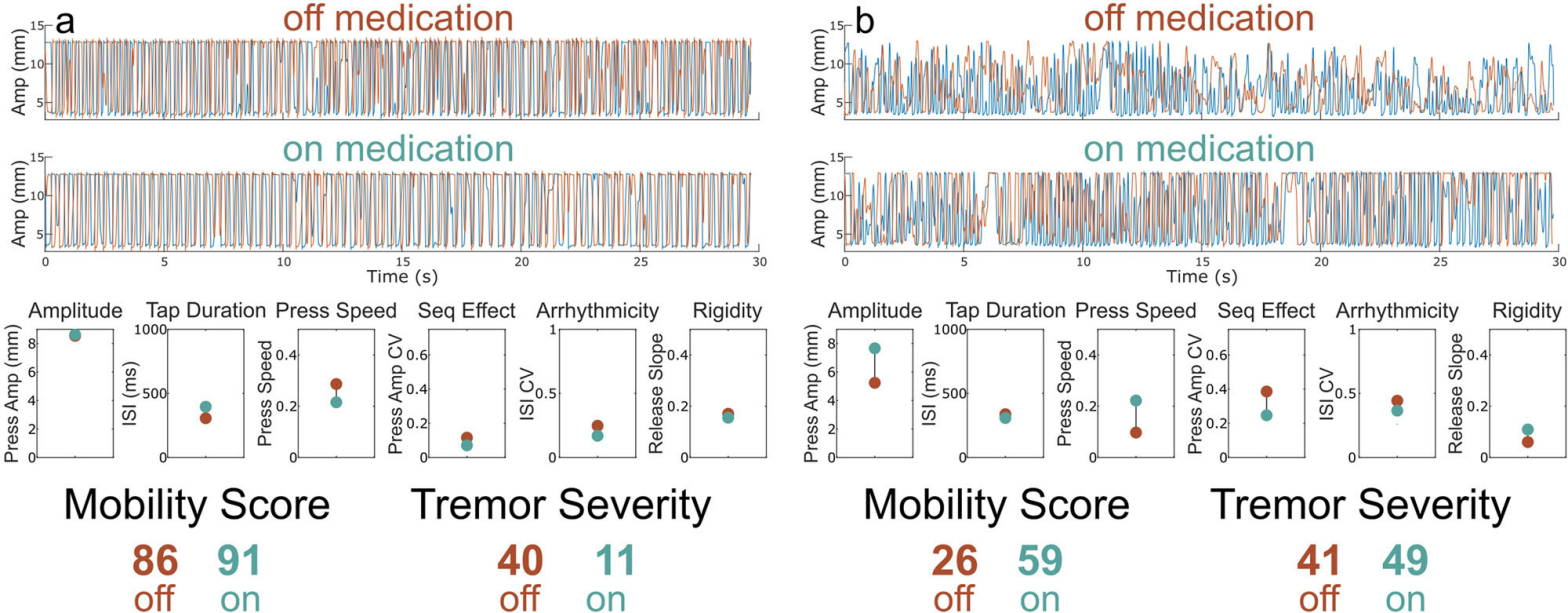
Examples of change in Tremor Severity Score off versus on medication. (Top) Raw QDG trace for the index and middle finger off and (Middle) on medication. (Bottom) Performance across each of the six key QDG metrics and the corresponding total Mobility Score and Tremor Severity Score with off medication depicted in red and on medication in green. Subtitles denote which aspect of movement is being measured. **a** Example of participant with high initial Mobility Score for voluntary tapping but the presence of some tremor which improves with medication. **b** Example of participant with low initial Mobility Score and a moderate amount of tremor off medication. Medication improves overall Mobility Score but does not improve tremor.

Figure 4a shows a QDG-RAFT trace from a tremor dominant PWP. Their performance off medication revealed full amplitude strikes and only mildly impaired voluntary tapping, as demonstrated by a Mobility Score of 86. However, this individual demonstrated tremor for 9% of the trial with an average amplitude of 7.1 mm. This corresponded to a Tremor Severity Score of 40. On medication, there was a small improvement in the already high Mobility Score (91) and a noticeable reduction in tremor, which was now only present for 3% of the trial; the Tremor Severity Score reduced to 11. Figure 4b shows a QDG-RAFT trace from a PWP whose tremor did not respond to medication. Off medication, they show significantly impaired voluntary tapping marked by low amplitude presses and loss of amplitude over time (i.e., sequence effect) resulting in a mobility score of 26. This individual also had tremor for 18% of the trial with an average amplitude of 5.7 mm, resulting in a Tremor Severity Score of 41. On medication, their Mobility Score improved to a 59 due to improvements in amplitude, press speed, and sequence effect. However, they still showed tremor for 20% of the task with a Tremor Severity Score of 49.

### Remote monitoring software

Designing a remote monitoring system requires a well-architected solution, putting the patient at the center. The system not only needed to easily transmit analyzed movement data from the patient and present it to the provider, but also instruct the patient in task performance; manage potential performance errors; collect, transmit, and present accurate and up-to-date therapy information; and support the provider in interpreting results to enhance clinical decision-making.

The mobile application is the central point of contact for the patient to interact with the device and inform providers about medication adherence and DBS settings. The mobile application was designed to run on Apple iPads and iPhones using the Stanford Spezi^23^ open-source project that provides a mechanism for user onboarding, account management, Bluetooth connectivity, medication tracking, and other reusable modules used in the QDG application. The Bluetooth connection is automatically paired to the device, the device state is shared with the application, and the patient is guided through the testing flow using videos, images, audio, and text. Specifically, patients are instructed as follows: “The goal of this task is to fully press and release the keys with your index and middle fingers in an alternating fashion. Do this as fast as possible and as consistent as possible. Try not to lift your fingers off the keys. You will begin the task when you hear the “Go” cue, and stop when you hear the “Stop” cue. Look forward at the iPad, and not down at your hand.” The task is self-paced and the application does not provide real-time feedback to the patient about their performance during task execution, with the exception of error detection. The application prompts the user to redo the task if a delayed start was detected or if the user only pressed down one of the keys within the first 5 seconds, avoiding a timely and delayed response from cloud processing. The application was constructed to support dynamic text sizes and a simple choice of three main actions (start a test, medication settings, and DBS settings) provides a streamlined user experience.

The design goal of the QDG-Care cloud processing architecture and QDG web dashboard was to provide a scalable solution that will eventually support a large number of patients, hospital systems, and research institutions. The system was designed using web service instances accessing a shared relational database and a scalable cloud storage bucket for the raw data upload from the mobile application. Incoming tests are queued to be processed by algorithm microservices pulling the information from the web service context and cloud storage buckets and processing the results asynchronously. The goal of this design is to ensure that data can be reprocessed if needed. At the same time, most interactions can be served using the insights generated using the algorithm, including the key extracted metrics and a graphical representation of the conducted tests as shown in the web dashboard. The EHR-integrated dashboard (Figs. 1 and 2) presents providers with insights about each patient, including an overview of the medications taken across different time scales (e.g., days and weeks), as well as an overview of the QDG metrics and medication adherence represented as time series data. Presenting the dashboard in the EHR, SMART on FHIR^24^ offers a convenient way to launch a web application within the context of a wide variety of EHR systems, including Stanford Medicine’s Epic instance. The data is available within the patient’s chart using a button which launches the web dashboard in a patient-specific representation, allowing a provider to immediately access the key data insights required to improve the patient’s medications, DBS settings, and overall treatment.

### In home remote QDG monitoring system

The protocol for the pilot clinical study assessing in-home remote monitoring using QDG-Care was designed to capture comprehensive motor data at least once per day over an initial period of 30 days. The study is ongoing, and the preliminary results for the first 8 participants out of 20 planned are reported below. Participants taking medication were asked to test twice per day when they were feeling at their worst “off” and best “on” state. The Centers for Medicare and Medicaid Services require that a remote therapeutic or patient monitoring test is done at least 16/30 days to be eligible for reimbursement and this was the primary outcome. In addition, the ability of QDG to track motor symptoms in a variety of use case scenarios was explored. See Methods for more information.

Compliance with monitoring using the QDG system was high with 8/8 participants (100%) performing at least one test for a minimum of 16/30 days. Furthermore, 7/8 participants (87.5%) performed at least two tests per day for at least 16/30 days of the testing period. The eighth participant was not on medication and was asked to test once per day. Participants reported missing an occasional test most often due to busy schedules, traveling or holidays, or technical difficulties while completing a test. All participants stated they would continue using the device given the opportunity.

Daily testing with QDG provided unique insights into how motor symptoms changed in response to medication, both within a given day and over the course of days to weeks, and how they progressed within the first month of diagnosis.

**Fig. 5:**
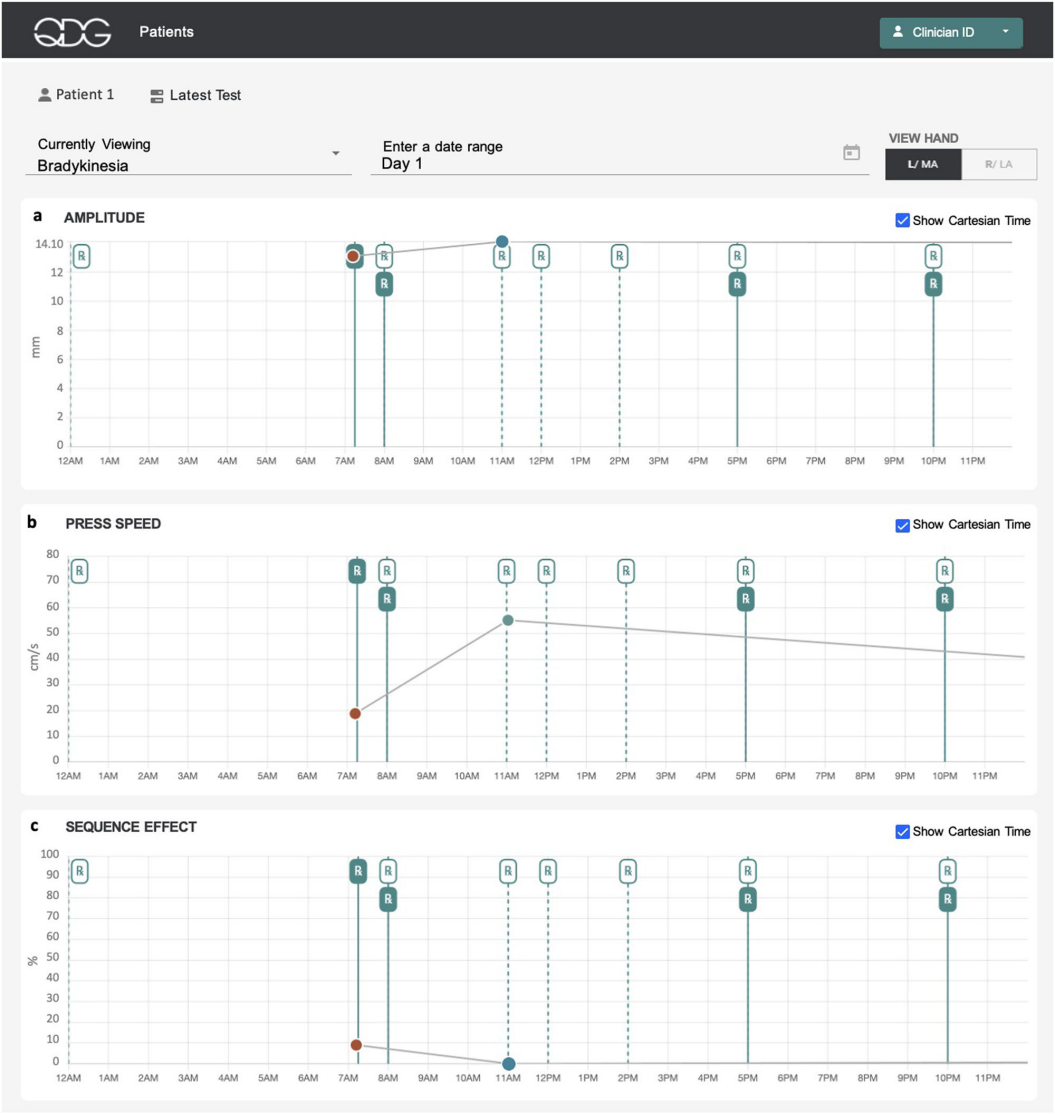
Depiction of QDG web dashboard of a single day of testing. The metrics of bradykinesia, specifically press amplitude (**a**), press speed (**b**), and sequence effect (**c**), are shown. On the day represented, the patient completed their “off “ test at 7 am and the “on” test at 11 am as denoted by the circular data points on the chart. The red circles correspond to QDG metric values that deviate from the range of metric values for healthy controls, while the green circles correspond to metric values that fall within the range for healthy controls. The ‘Rx’ markings designate the prescribed Parkinson’s medications for the participant, where ‘Rx’ markings with the dotted line indicate the participant’s scheduled prescription timepoints as decided by their neurologist, while filled ‘Rx’ markings with a solid line indicate when a participant actually took their medication.

QDG reflected the effect of one or two doses of medication and monitored adherence to time critical medication dosing. Figure 5 depicts a representative participant’s QDG metrics of bradykinesia from a single day of testing. The participant had PD for over 10 years and endorsed wearing off symptoms and occasional dyskinesias. They were prescribed dopaminergic medication six times a day, (Fig. 5 Rx dotted lines) and stated that they felt at their worst off state in the early morning before taking medication and at their best on state in the late morning. The participant’s metrics of bradykinesia improved after they took medication and the values moved from the abnormal to normal range, Fig. 5a–c. This demonstrates that QDG metrics reflect changes in motor symptoms in relation to medication intake over the course of a single morning. The daily chart view monitored the time they took medication compared to the time it was prescribed to gauge their ability to adhere to the frequent dosing schedule.

**Fig. 6:**
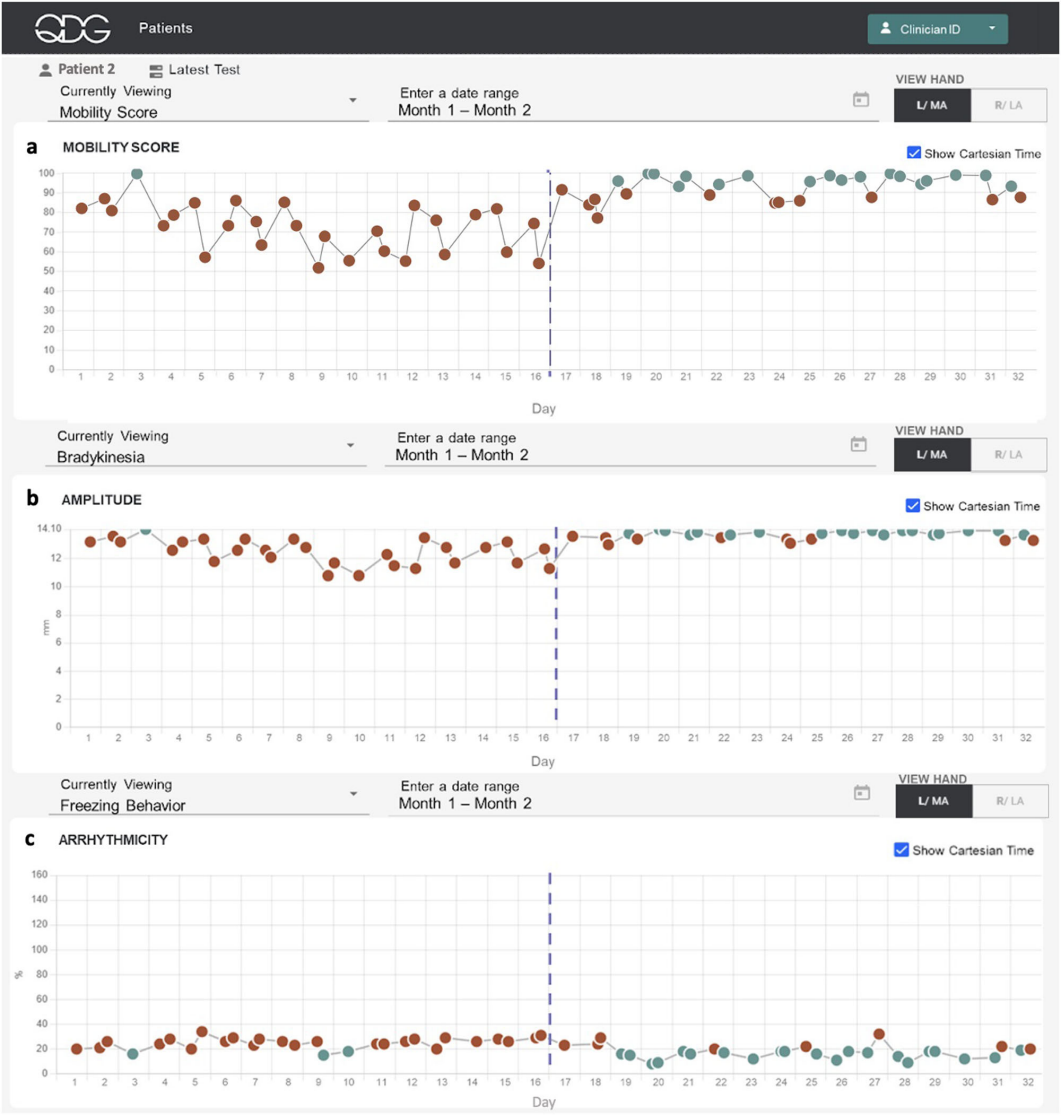
Depiction of QDG web dashboard of 30 days of testing. Metrics displayed are (**a**) Mobility Score, (**b**) Press Amplitude in mm and (**c**) Arrhythmicity in %. Red circles—abnormal values, green circles—normal values. The vertical dashed lines mark the day of a change in therapy.

QDG-RAFT also provided insight into how small changes in medication dosing can impact motor symptoms on a longer-term scale. Figure 6 presents remotely obtained QDG metrics for one participant’s left hand over a 30-day period. This participant, in the early stages of PD, experienced gait as the most bothersome symptom. Their MDS-UPDRS III gait sub-score improved from 2 to 1 after starting a regimen of immediate-release carbidopa - levodopa (CD/LD) 25/100, one tablet taken three times a day. This was matched by their and their family’s perception that their gait and facial expression improved, although they did not notice any difference from individual doses, as is typical in early stage PD. The participant took the first medication dose at 6:00 am. The second dose was taken at approximately 9:30 am, preceding a daily walk roughly 30 min later. They decided to try taking an extra dose of CD/LD at their second dose to see if it would improve their walking further (dashed line Fig. 6). This resulted in noticeable improvements in the QDG metrics: their QDG-Mobility score, bradykinesia-amplitude, and arrhythmicity scores for the left hand improved from abnormal to within the normal range. This improvement was accompanied by a reduction in day-to-day variability, suggesting a more consistent response to the extra dose of medication.

**Fig. 7:**
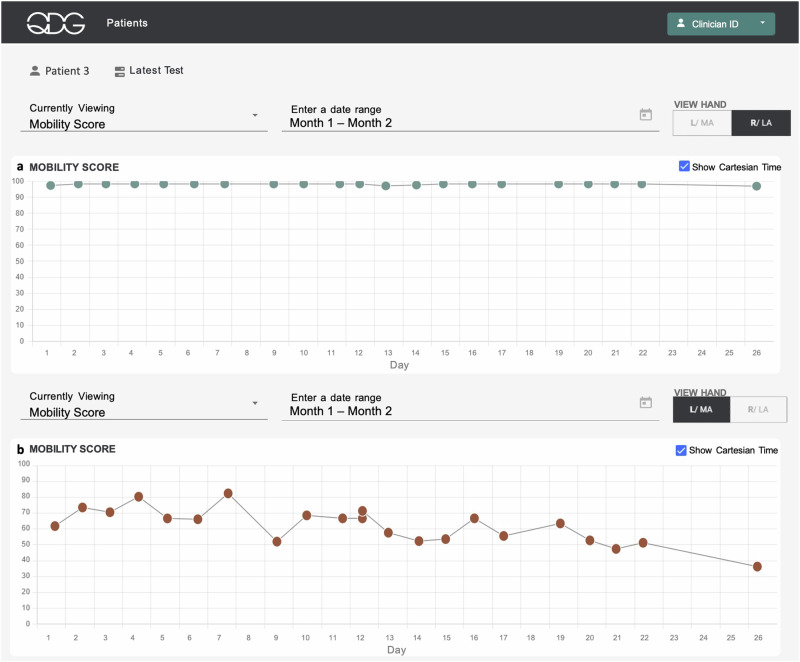
QDG web dashboard tracking QDG Mobility Score over time. **a** (Right Hand) and **b** (Left Hand) illustrate the QDG Mobility Scores for a participant tracked over 26 days following PD diagnosis and before initiation of therapy. Red circles—abnormal values, green circles—normal values.

Figure 7 illustrates the progression of Parkinson motor disability on the more affected side within the first month after receiving the clinical diagnosis of probable PD and before any treatment was initiated. The participant had presented on Day 1 of Fig.7 stating a history of about a year of intermittent left hand tremor only while walking and no other complaints. Their initial exam revealed emergent left hand tremor only when walking and mild left sided bradykinesia and rigidity. The MDS-UPDRS III score was 16, with clear asymmetry between left and right sides. They were enrolled in the at-home study for a month while supportive laboratory and nuclear medicine (DaTScan) tests were performed. Throughout the study period, the participant’s right (lesser affected) hand consistently maintained a QDG Mobility Score of 100 (maximum score, Fig. 7a). In contrast, the left (more affected) hand showed a significant decline in the QDG Mobility Score over time (Fig. 7b, linear regression: β = −1.18 [−1.70 −0.67], ${R}^{2}$=−0.74, *p* = 1.2e−4). The DaTScan revealed diminished uptake of the tracer in the bilateral putamina, worse in the right hemisphere.

## Discussion

The development of QDG-Care represents a significant advancement in the management of PD. QDG is a novel validated technology for objectively measuring all the motor signs of PD and delivers it to the provider in real time. Transformation of QDG technology into a remote system followed an iterative design approach centered on patient and provider needs. The mobile application guides the patient through data collection and RAFT performance on the portable, Bluetooth-enabled QDG KeyDuo, which accurately and precisely measures the amplitude and timing of finger movements. The QDG PRECISE algorithm performs real-time, automated analysis of data from the device and extracts quantitative metrics for all the motor signs of PD, which have previously been validated. The web application presents providers with flexible and comprehensive views of symptom severity, including the newly developed QDG Mobility and Tremor Severity Scores.

A crucial enabler of the system for providers is the transformation of these metrics to actionable results, by contextualizing them not only relative to healthy controls but in regards to critical therapeutic information. The availability of various data viewing formats, from detailed single tests to aggregated data over time, aids in identifying trends and changes in motor symptoms. These insights provide clinicians the ability to personalize care, even in remote settings. In the remote clinical trial reported here, QDG technology enabled the provider to monitor motor function in an individualized manner over time and to make data-informed decisions regarding PWP’s responses to adjustments in complex, time-critical medication regimens. The current study supports clinical uptake of QDG from: the demonstration of 100% compliance in the pilot trial with testing at least 16/30 days in a month, which is required for remote monitoring reimbursement, the broad range of use cases that QDG covers, and the accessibility to the platform and dashboard through the EHR. Integration into the EHR enables more convenient access and the ability to view results in a patient-specific context. To our knowledge this is the first comprehensive remote monitoring system for PD that provides results in real time and which are viewable from the EHR.

QDG-Care will enhance telemedicine with a direct clinical application to remote monitoring. Remote monitoring using QDG will drive both personalization of care and expanded access to high quality care, as well as enhance the evaluation of potential therapeutics.

The QDG Mobility Score was developed to provide a statistically-driven measure of an individual’s overall voluntary motor behavior by comparing the presentation of QDG metrics to the performance of age-matched healthy controls. The QDG Mobility Score is similar to the MDS-UPDRS III in that it provides an overall score composed of various sub-scores. However, it differs in that the MDS-UPDRS III reflects symptom severity, whereas the goal of the QDG Mobility Score is to reflect movement proficiency. The QDG Tremor Severity Score complements the QDG Mobility Score by providing comprehensive insight into the severity of involuntary tremor, derived from percent duration of tremor and tremor amplitude^18^. While the individual metrics provide high-resolution insight into individual symptoms, the QDG Mobility and Tremor Severity Scores serve as comprehensive measures of symptom severity and overall motor behavior presented through easily interpretable yet informative values, which is pivotal for informed clinical decision-making. For example, an improvement in mobility score but little change in the tremor severity score on medication compared to off, Fig. 4b, is indicative of medication refractory tremor which is valuable insight for deep brain stimulation (DBS) evaluations. The standard clinical rating scale typically used for these evaluations, the MDS-UPDRS III, does not reflect such insight in an easily digestible format. This could have meaningful implications around procedure eligibility and insurance coverage for the procedure. On the other hand, improvements in tremor severity with small changes in the mobility score suggest efficacy of a therapy modality for tremor but not for voluntary movement. Therefore, the distinct evaluation of voluntary and involuntary movements gives the provider comprehensive, high-level yet clinically relevant information that could guide and improve clinical decision making.

Preliminary results from the pilot study evaluating remote monitoring with QDG not only demonstrated that patients were highly compliant with testing, but that it provided clinical value in a variety of scenarios. The system supplied critical information that would likely have been missed in standard clinical practice. A single day of testing with QDG demonstrated a marked improvement in the participant’s motor symptoms in response to one or two doses of medication, while daily testing over a month demonstrated that a participant-initiated addition of a single tablet of carbidopa/levodopa (25/100) resulted in persistent improvements in QDG metrics of motor function, moving from the abnormal to normal ranges, Fig. 6. Remarkably, daily monitoring over the month immediately following a diagnosis of PD demonstrated a significant decline in the QDG Mobility Score on the more affected side while the score remained at its maximum (100) on the lesser affected side, Fig. 7. This is a unique demonstration of a significant decline in motor function within the first month after a clinical diagnosis of PD before treatment was started.

In all cases, QDG technology enabled the provider to monitor motor function in an individualized manner over time and to make data-informed decisions regarding PWP’s responses to adjustments in complex, time-critical medication regimens. This approach not only enhances patient care but also paves the way for personalized treatment strategies in PD management. Furthermore, demonstrated compliance with testing at least 16/30 days in a month satisfies reimbursement requirements, which will facilitate clinical uptake. The pilot clinical study is ongoing and additional use cases, such as for DBS programming, are being explored.

QDG-Care is capable of providing additional clinical value outside of remote monitoring. DBS evaluations often require that patients travel long distances in the off state to undergo motor testing in clinic and then must stay for additional on testing. With QDG technology, DBS evaluations can be completed quickly and in the comfort of the patient’s home. It can also be used in non-neurology clinics or in the emergency room (ER) to speed up treatment recommendations by the consultant neurologist, who may not be on site or available. The inpatient length of stay (LOS) is longer in PWP compared to age-matched controls due to mismatched medication schedules from their outpatient regimens^25^. PWP enrolled in QDG-Care will have a seamless transition of medication schedules from the outpatient to inpatient settings that will shorten LOS and improve outcomes. The use of QDG in the ER and inpatient settings will reduce the current economic burden of care in PD, which is currently over $51 Billion annually^9^.

High resolution metrics combined with reduced complexity of patient monitoring using the remote system will also allow rapid “Go-NoGo” decisions of potential therapeutics, requiring fewer participants at a lower cost and allowing remote recruitment and assessment. The flexible architecture of the system allows the incorporation of multiple algorithms and will allow researchers to perform validations of new and innovative algorithms within a high-quality and high-resolution dataset. We envision QDG as a future platform for validated PD-related digital biomarkers that can be used to perform population-wide insights into procedures and quantifiable metrics of PDWs in different stages of the care journey.

Current limitations include the EHR integration focus specific to the Stanford Hospital Epic instance. While SMART-on-FHIR integrations and single-sign-on mechanisms are standardized, integrating the system and workflows into other EHR systems will require additional work and provide additional insights into shaping the project design. In this pilot study, the study staff conducted in-person visits to set up participants with the system in their homes and provide in-person assistance with troubleshooting the system. Therefore, initially we focused on participants within the local region. We plan to expand the geographical area of recruitment for a planned cohort of 20 participants for the remainder of the pilot trial. Finally, the system does not yet include measurements of medication-induced dyskinesia or speech impairment. However, with the currently designed system architecture, these measurements may be added at a later stage of development.

In conclusion, we have introduced QDG-Care, a fully integrated connected care platform consisting of a high precision, Bluetooth-enabled digitography device (KeyDuo), a patient-facing mobile application with local processing, a HIPAA-compliant cloud web service and customized algorithm (PRECISE), a provider dashboard, and EHR integration. The foundational QDG technology yields validated quantitative metrics for all the motor signs of PD in real-time. Transformation of QDG technology into a remote system followed an iterative design approach centered on patient and provider needs. Preliminary results from the remote monitoring trial supported clinical uptake with full compliance with requirements for remote monitoring reimbursement. QDG-Care represents a significant advancement in the management of Parkinson’s disease and will enhance telemedicine with a direct clinical application to remote monitoring. Remote monitoring using QDG technology will drive both personalization of care and expanded access to high-quality care, as well as enhance the evaluation of potential therapeutics in clinical trials. This approach also paves the way for personalized treatment strategies in PD management in clinic and inpatient environments.

## Methods

Through clinical practice and research, the principal investigator has interfaced with numerous PWP, other healthcare providers, and industry partners, and the earliest user needs for a comprehensive, objective, and remote system for monitoring motor symptoms were derived from these interactions. The research team also conducted semi-structured interviews with 22 PWP to better understand remote symptom tracking needs generally and with 34 PWP to better understand remote symptom tracking with QDG specifically. Finally, the team received input through semi-structured interviews with a national sample of 7 providers and from a local focus group of 6 providers treating PWP. Providers included general neurologists, movement disorders specialists, nurse practitioners, and nurses.

### QDG KeyDuo Design

The design of the re-engineered digitography device was informed by patient user needs and preferences, devices used in previous research, and technical requirements of the algorithm. In the semi-structured interviews to better understand needs and preferences around remote symptom tracking using QDG technology, PWP provided input on locations of use, portability, storage, connectivity, appearance, and feel of the device.

The QDG-RAFT task was originally developed on a musical instrument digital interface (MIDI) keyboard, was subsequently employed on an engineered set of levers that were part of the At Home Testing Device developed in collaboration with Intel’s Digital Health Technology Group, and was finally developed in a lab prototype that has been used in our research for over 12 years^11,12,14–19^. The mechanical and signal characteristics of each device were considered in conjunction with algorithm needs to accurately and precisely detect the amplitude of lever presses and releases and the timing of critical phases of lever presses and releases. Temporal keypress position data from the KeyDuo device are measured with a sampling rate of 200.96 Hz by the on-board microcontroller. Raw data (~180 KB per hand) are transmitted via Bluetooth to the mobile application and then on to the web service for further processing.

### Development of Mobility Score and Tremor Severity Score

51 individuals diagnosed with Parkinson’s disease (70.5% male, 29.5% female) and 42 healthy controls (47.6% male, 52.4% female) were included in the dataset. There was a significant difference in age between the two cohorts (PD Average Age: 67.7 ± 7.1 years vs. HC Average Age: 60.0 ± 9.0 years, *p* = 3.75e−5). The average disease duration of the individuals with PD was 7.7 ± 6.5 and the total MDS-UPDRS III score off therapy was 22.9 ± 12.5 and on therapy was 15.5 ± 7.1. Data was collected in the Stanford Human Motor Control and Neuromodulation Laboratory. PWP performed RAFT in the on and off-therapy state. For the off-therapy state, long- and short-acting medications were withdrawn over 24 to 48 and 12 h, respectively. For patients with DBS, stimulation was turned off for at least 15 min before testing. For the on-therapy state, participants were asked to take their Parkinson’s-related medication as usual. All participants gave written informed consent, which was approved by the Stanford University Institutional Review Board (eProtocols 30689, 30880, and 13917).

All participants were asked to perform a 30-second repetitive alternating finger tapping task with their index and middle fingers on tensioned, engineered adjacent keys on a digitography device, which senses the amplitude displacement and timing of key strikes. The instructions were to press and release each key in an alternating pattern as fast and regularly as possible. They were instructed to attempt to press and release the keys completely. Participants performed the task with each hand. Individuals with Parkinson’s disease also performed the motor scale of the Movement Disorders Society-Unified Parkinson’s disease rating scale III (MDS-UPDRS III).

A customized detection algorithm was used to determine the specific states in the cycle of finger movement. Several metrics were calculated from these cycles: 1) Press amplitude, 2) Press amplitude coefficient of variation (CV: standard deviation/mean), 3) Inter-strike interval (ISI: time to complete one cycle of finger movement), 4) Inter-strike interval CV (ISI CV), 5) Release slope (i.e., ratio of the amplitude of the key release compared to duration of release), 6) Press speed (i.e., ratio of the amplitude of the key press compared to duration of press), 7) Dwell time (i.e., duration at bottom of the press), and 8) Rest Tremor % (RT%: percent of QDG trace with rest tremor). The average for each of these metrics was computed for each finger and then averaged across fingers for each hand.

**Fig. 8:**
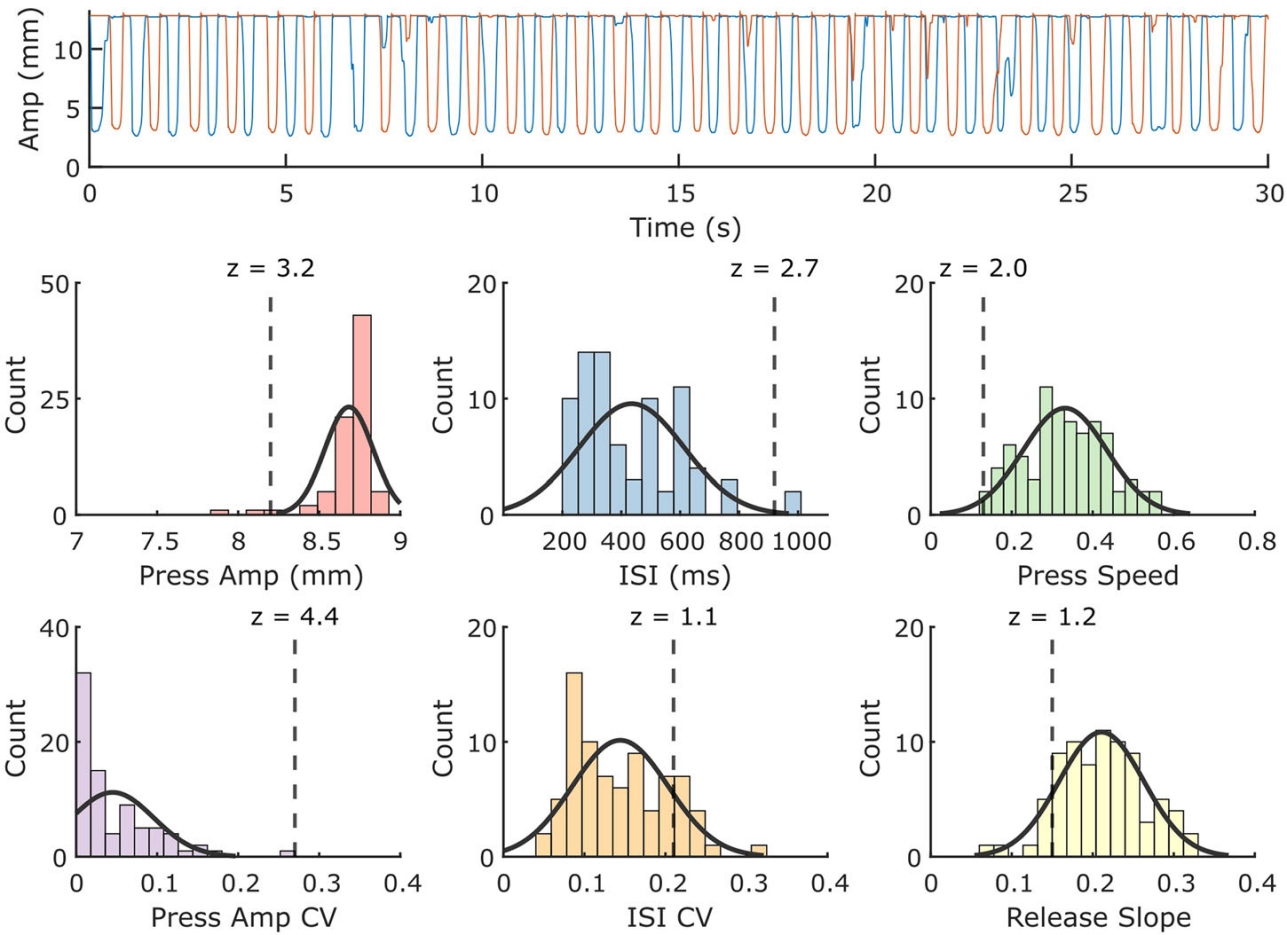
Visualization of Mobility Score Calculation. (Top) Example of raw QDG trace from index and middle finger from a PD patient. (Bottom) Histograms and accompanying normal distribution fits for data from the healthy control cohort for each of the main QDG metrics. The vertical dashed line represents the value from the PD trace and its corresponding Z-score.

The QDG Mobility Score consisted of the six QDG metrics used to determine the motor signs in PD: ISI, ISI CV, Press Speed, Press Amplitude, Press Amplitude CV, and Release Speed. A distribution for each of these metrics was created based on healthy control data. We employed a linear mixed effects model to assess the correlation between metrics and age. Age exhibited a strong and statistically significant impact on Press Amplitude CV, Release Slope, and Press Speed. To account for the influence of age, we conducted adjustments for these metrics by assuming a linear age effect. The adjustment formula utilized was as follows:

1 $${Metri}{c}_{{Adjusted}}={Metric\; Value}+{slope}\times ({Age}-{mean\; cohort\; age})$$

where the slope corresponds to the determined slope from the linear mixed effects model specific to each metric, and the mean cohort age was set at 60. After adjusting for age, a z-score was assigned for each metric for incoming data from a PWP (Fig. 8).

The overall Mobility Score was calculated based on the average observed z-scores across metrics using the following equation:

2 $${Mobility\; Score}=100-14\times \frac{1}{n}{\sum\limits_{i=1}^{n}}{z}_{i}$$

where *z* is the observed z-score for a given metric for the data point and *n* represents the total number of QDG metrics. The Mobility Score ranged from 0 to 100, with 100 representing perfect mobility. The sign of the z-score was flipped in cases where a negative z-score would indicate worse performance (e.g., Press amplitude, press speed, and release slope). Negative values (i.e., above average performance) were capped at 0 so that above average performance in any metric did not artificially inflate the overall score. Given the tightly clustered distribution of press amplitudes around full amplitude in healthy controls (mean: 8.67 ± 0.12 mm), the calculation of the mobility score for trials that had lower press amplitudes was primarily dominated by a high absolute press amplitude z-score value, diminishing the impact of other metrics. Therefore, for trials with an absolute press amplitude z-score value greater than 10, which corresponded to a press amplitude below 7.5 mm, the press amplitude z-score value was transformed using a power law equation to progressively decrease the impact of lower press amplitude values on the overall mobility score. The press amplitude z-score was adjusted based on the following equation:

3 $${z}_{{transformed}}=A\times {\left(\left|{z}_{{PressAmp}}\right|\right)}^{k}$$

where $A$ = 3.2 and $k$ = 0.495. The equation parameters were defined to ensure that the maximum transformed z-score value did not exceed 20. A continuous transition of transformed values was maintained at a press amplitude z-score of 10, mitigating any potential discontinuities in the transformed values.

The Tremor Severity Score used a previously validated XGBoost Classifier to classify the presence of tremor on a per-strike basis based on the QDG metrics described above^18^. After identifying which strikes were tremor, the average amplitude of the tremor strikes was extracted. The Tremor Severity Score was then based on a combination of the percent duration of tremor in the trial and the average amplitude of tremor using the following equation:

4 $${Tremor\; Severity}=\frac{\, \% {Rest\; Tremor}+100\times \left(\frac{\frac{1}{n}{\sum }_{i=1}^{n}{{Amp}}_{i}}{{{Amp}}_{{Max}}}\right)}{2}$$

where *Amp* is the observed press amplitude for a given tremor strike, *n* represents the total number of identified tremor strikes, and ${{Amp}}_{{Max}}$ is the max possible amplitude on the QDG device. For trials with a low percent tremor (i.e., < 10%), we transformed the amplitude component in order to suppress its impact on the overall tremor severity score using the following equation:

5 $${{RT}}_{{amp}}={A}^{k( \% {Rest\; Tremor})}+b\,$$

where $A$ = 4, $k$ = 0.05, and $b$ = -1 and ${{RT}}_{{amp}}$ is the transformed normalized rest tremor amplitude.

The thresholds for determining whether a QDG metric was within or outside of the expected range was determined based on the distribution of Healthy Control values for each metric. The threshold of metrics for which lower values are reflective of progressively worse performance (Press Amplitude, Release slope, Press Slope, Taps per minute, Mobility Score) was defined as the 25th percentile value of healthy control data for that metric. Subsequently, the threshold of metrics for which higher values are indicative of worse performance (ISI CV, Press Amplitude CV) was established as the 75th percentile value of healthy control data for that metric.

Statistical analyses were run in MATLAB (version 9.9, Mathworks, Natick MA) and Spyder Integrated Development Environment (IDE). A Linear mixed effects model with a fixed effect of age and a random intercept for subjects was used to evaluate the impact of age on each of the QDG metrics. A two sample *t*-test was used to evaluate the difference in ages between control participants and PWP. To assess the the association between hemibody MDS-UPDRS III scores and the QDG Mobility Score, a linear mixed effects model was used with fixed effects of the hemibody MDS-UPDRS III scores and Mobility Score, with a random slope of therapy condition and random intercept for subject. A linear regression was performed to evaluate the change in mobility score over time. Significance was set at *p* < 0.05.

### Remote monitoring software

The design of the remote monitoring software system was informed by patient and provider user needs identified through semi-structured interviews and focus groups as described above. The system follows software engineering best practices. The mobile application and Bluetooth protocol implementation are automatically tested and validated on each software release to participants. Data between subsystems is transferred using a Bluetooth Low Energy protocol to communicate between the device and mobile application and HTTP-based RESTful APIs secured by industry-standard encryption in transit. The web service architecture is modeled so different versions of the QDG algorithm and other external algorithms can be executed in separate compute instances, allowing the execution of longer tasks and queuing mechanisms for incoming testing data. Frequently accessed data is stored in a Microsoft SQL Server database, while data read less frequently, such as the raw test measurements, are stored in cloud storage buckets. The web dashboard uses standard web application development techniques and communicates with the web service using a RESTful API. The dashboard can be surfaced in the EHR system using a SMART-on-FHIR launch mechanism or can be presented as a standalone web interface that is accessible to Stanford clinicians and staff.

### At-home study

Eight individuals (3 female, 5 male) with Parkinson’s disease were recruited from the Stanford University Movement Disorders Clinic for the pilot remote QDG at-home study. Participants were 71 ± 8.1 years old and had a disease duration of 7.3 ± 5.0 years (based on the year of diagnosis) or 8.4 ± 4.9 years (based on the year of symptom onset). Participants included individuals who were being evaluated for or had a diagnosis of PD and gave written informed consent, approved by the Stanford Institutional Review Board (eProtocol 60883), to participate in the study. Individuals less than 18 years old, unable to provide informed consent, unable to follow directions, or unable to perform the task due to pain and/or musculoskeletal injury or disease were excluded. This is an ongoing study with a planned enrollment of 20 participants.

The at-home study was designed to evaluate the feasibility of using the QDG system to remotely track movement symptoms in Parkinson’s disease for at least 16/30 days, a requirement of CMS for reimbursement of remote patient or therapeutic monitoring. Participants taking medication were instructed to test twice a day; once in the morning before they took their medications (“off”) and once later in the day when they were feeling their best (“on”). During the initial visit, participants completed a baseline QDG-RAFT task and responded to questions about their symptoms and disease state. Trained members of the research team then assisted participants in finding a suitable location in their home at which to perform QDG-RAFT. The task must be performed in a seated position with the system placed at a reachable distance on a flat, stable surface. Research personnel reviewed the user guide with the participant, which included information on how to set up and use the mobile application, device, and system as a whole, device care and handling, inputting medication and DBS settings, navigating initial therapy screens, correct arm and hand placement, and how to complete a test. After completing training, members of the research team observed the participant complete a task on both hands on their own. The 30-day testing period began after completion of the initial visit. Weekly check-ins were conducted either in-home or via video conferencing once a week for 4 weeks with trained members of the research team. At each weekly check-in, a member of the research team completed questionnaires with the participant regarding testing compliance, perceived device or mobile app functionality and usability, as well as observing the participant complete the QDG-RAFT task. The participant also completed a check-in with the study doctor during the 30-day trial in order to review their QDG-RAFT data and progress. Upon completion of the 30-day period, trained members of the research team visited the participant’s home to complete their final weekly check-in, which included an additional exit interview where participants were asked if they would continue using the device if given the opportunity.

## Data Availability

The datasets used and/or analyzed during the current study are available from the corresponding author [HBS] on reasonable request for research studies with a defined scientific question and plan pertaining to the use of the data.

## Code availability

The underlying code for this study is not publicly available but may be made available to qualified researchers on reasonable request from the corresponding author.
